# Preparation of Core-Shell Rare Earth-Doped Upconversion Nanomaterials and Simultaneous Detection of Two Pesticides in Food

**DOI:** 10.3390/foods11101485

**Published:** 2022-05-19

**Authors:** Wenbo Zhu, Lingyan Zhao, Jingyi Jin, Yang Song

**Affiliations:** 1 Tianjin Key Laboratory of Animal and Plant Resistance, College of Life Sciences, Tianjin Normal University, Tianjin 300387, China; 1910170006@stu.tjnu.edu.cn (W.Z.); 2010170003@stu.tjnu.edu.cn (L.Z.); 2110170005@stu.tjnu.edu.cn (J.J.); 2 State Key Laboratory of Food Nutrition and Safety, College of Food Science and Engineering, Tianjin University of Science and Technology, Tianjin 300457, China

**Keywords:** upconversion nanomaterials, core-shell structure upconversion nanomaterials, imidacloprid, clothianidin, food

## Abstract

Under the excitation of a 980 nm excitation light, the fluorescence signals of the synthesized core-shell NaYF_4_:Yb@NaYF_4_:Ho and monolayer NaYF_4_:Yb,Tm upconversion nanoparticles (UCNPs) were simultaneously detected at 656 and 696 nm, respectively. The two upconversion materials were coupled with anti-clothianidin and anti-imidacloprid monoclonal antibodies by the glutaraldehyde cross-linking method as signal probes. Imidacloprid (IMI) and clothianidin (CLO) could compete with antigen-conjugated amino Fe_3_O_4_ magnetic nanomaterials for binding to signaling probes, thus establishing a rapid and sensitive fluorescent immunoassay for the simultaneous detection of IMI and CLO. Under optimal conditions, the limits of detection (LOD, IC_10_) and sensitivity (IC_50_) of IMI and CLO were (0.032, 0.028) and (4.7, 2.1) ng/mL, respectively, and the linear assay ranges were at 0.032–285.75 ng/mL and 0.028–200 ng/mL, respectively. Immunoassay did not cross-react significantly with other analogs. In fruits and vegetables such as apples, oranges, peaches, cucumbers, tomatoes and peppers, the mean recoveries of IMI and CLO ranged from 83.33% to 115.02% with relative standard deviations (RSDs) of 1.9% to 9.2% and 1.2% to 9.0%, respectively. Furthermore, the results of the immunoassay correlate well with the high-performance liquid chromatography method used to detect the actual samples.

## 1. Introduction

Neonicotinoids (NEOs) are the fourth largest class of pesticides after organophosphates, carbamates and pyrethroids. They have the characteristics of high compatibility with the environment and no cross-resistance with other traditional pesticides [1]. As of 2012, more than 120 countries have registered the use of neonicotinoid pesticides. IMI (36.9%) and CLO (14.3%) account for more than half of the total share of these pesticides [2]. However, such pesticides were constantly detected in food, soil and groundwater, which brought great potential safety hazards to human health and the ecological environment [3,4] and had a certain negative impact on pollinators such as bees [5,6]. They have also had lethal effects on aquatic and terrestrial invertebrates [7,8]. In addition, IMI and CLO have also had potential toxic risks such as hepatotoxicity, neurotoxicity and genotoxicity, which have attracted wide attention from countries around the world [9,10].

In response to the problems of the above neonicotinoid pesticides, China’s GB 2763-2021 “National Food Safety Standard Food Maximum Residue Limits for Pesticides” [11] stipulated that the maximum residue limits (MRLs) of IMI and CLO in fruits and vegetables should be 0.05–5 mg/kg and 0.01–2 mg/kg. The EU stipulated that the MRLs of IMI [12] and CLO [13] should be 0.05–5 mg/kg and 0.01–1.5 mg/kg in fruits and vegetables, respectively. In addition, the United States [14], Canada [15] and other countries have also banned and restricted such pesticides. The current detection techniques for neonicotinoid pesticides include liquid chromatography [16], liquid chromatography-tandem mass spectrometry [17], and enzyme-linked immunosorbent assay [18]. Li Dongpo et al. [19] established a high-performance liquid chromatography-tandem mass spectrometry method for the determination of IMI and emamectin-benzoate residue in honeysuckle, with quantitative limits of 2.5 and 10 μg/kg, respectively, which provides a theoretical basis for the detection of such pesticides in traditional Chinese medicine. But its pretreatment is relatively cumbersome and time-consuming. Watanabe et al. [18] used the enzyme-linked immunosorbent assay (ELISA) to detect dinotefuran, IMI and CLO in leeks. The average recovery of spiked samples was 85–113%, and the relative standard deviation was less than 15%. However, ELISA has the disadvantages of many operation steps and false-positive results. Hua et al. [20] established a fluorescence immunoassay for the detection of IMI by using the synthesized NaYF_4_/Yb,Er UCNPs. The IC_50_ and detection limit (IC_10_) of IMI were 14.59 and 0.74, respectively. It can only detect a single target, which has certain limitations. Instrumental method detection requires professional operation and consumes a lot of solvents. The enzyme-linked immunosorbent assay requires multi-step incubation and washing procedures, which are cumbersome and time-consuming, and cannot meet the rapid monitoring of the market. Therefore, it is of great practical significance to establish a sensitive, efficient and convenient method for the detection of new nicotine pesticides.

Upconversion nanoparticles (UCNPs) could emit strong visible fluorescence under the excitation of a 980 nm light source and had the advantages of low toxicity, good chemical stability, a narrow emission band gap, and low fluorescence background interference [21,22]. However, there was still the problem that the surface is prone to fluorescence quenching. Core-shell structured upconversion nanomaterials formed by coating the core with one or more layers of materials may solve the problem [23] since the shell deposition may reduce the quenching effect caused by surface defects such as the suspension key and vibration groups and thus, effectively improve the luminous efficiency [21]. In addition, by rationally assigning the spatial position and relative concentration of the activator, precise upconversion luminescence color adjustment could also be achieved [24,25].

At present, NaYF_4_:Yb,Er, NaYF_4_:Yb,Tm and other UCNPs based on NaYF_4_ are the research focus in the field of nanomaterials. Excited by 980 nm light, NaYF_4_:Yb,Er and NaYF_4_:Yb,Tm emit green light and blue light at 540 nm and 470 nm, respectively, but the penetration depth of these two lights is small [26]. The light wavelength applied to deep-seated imaging of biological tissues is usually more than 600 nm, and the corresponding emitted light is red. At present, the UCNPs of Yb/Er and Yb/Tm co-doped systems are the main research objects of red-emitting materials, and there is little research on Yb/Ho system. Therefore, the study of Yb/ Ho co-doped UCNPs emitting red light can expand the application of biomarkers and biological imaging, which has important scientific significance and practical value. Research on core-shell structure upconversion materials is still in its infancy, and although we found many research papers in this area, there were few papers applying it to immunoassay detection. In this study, a new core-shell nanomaterial NaYF_4_:Yb@NaYF_4_:Ho was introduced into immunoassay for the first time, and a fluorescence immunoassay method based on magnetic separation and upconversion nanoparticles was established. This method has high sensitivity and accuracy and can be used for the simultaneous determination of IMI and CLO in food.

## 2. Materials and Methods

### 2.1. Materials and Reagents

IMI, CLO, acetamiprid (ACE), thiacloprid (THI), nitenpyram (NIT), dinotefuran (DIN), thiamethoxam (THX), ytterbium chloride, yttrium chloride, thulium chloride, holmium chloride, ammonium fluoride, oleic acid, cyclohexane, 3-aminopropyltriethoxysilane (APTES, 98%), tetraethyl orthosilicate (TEOS, 98%) were purchased from Adamas (Shanghai, China). Argon was purchased from Air Liquide Co., Ltd. (Tianjin, China). Bovine serum albumin (BSA, 98.0%) was purchased from Merck, Darmstadt, Germany. IMI antigen, CLO antigen, anti-IMI monoclonal antibody and anti-CLO monoclonal antibody were purchased from Shandong Landu Biotech-nology Co., Ltd. (Shandong, China). Anti-IMI monoclonal antibody and anti-CLO monoclonal antibody were purchased from Shandong Landu Biotechnology Co., Ltd. (Shandong, China). BCA (bicinchonininc acid) protein quantification kit was purchased from Solarbio (Beijing, China). Monodispersed Magnetite Microspheres (MNPs) were purchased from Beijing Baseline Co., Ltd. (Tianjin, China). The nanoparticle morphology and size of UCNPs were determined by FEI TECNAI G20 transmission electron microscopy (TEM, FEI Company, Hillsboro, OR, USA). Fourier transform infrared spectroscopy (FTIR) of UCNPs was determined by an FTIR spectrophotometer (Perkin Elmer, Waltham, MA, USA). Powder X-ray diffraction (XRD) measurements of UCNPs were performed by an AXIS-ULTRA-DLD instrument (Millipore, New York City, NY, USA). The TEM, FTIR and XRD characterizations of UCNPs were determined by the School of Chemistry, Tianjin Normal University. The fluorescence intensity of the upconverting nanoparticles was detected by an F-2500 spectrofluorometer equipped with a 980 nm laser exciter (Shimadzu Corporation, Kyoto, Japan). The high-performance liquid chromatograph (HPLC) was performed by Thermo Fisher Scientific, Waltham, MA, USA. All chemicals used were analytical grade.

### 2.2. Preparation of Upconversion Nanomaterials

#### 2.2.1. Preparation of NaYF_4_:Yb@NaYF_4_:Ho UCNPs

The NaYF_4_:Yb@NaYF_4_:Ho core-shell structure upconversion material was prepared based on a high-temperature thermal decomposition method. The total mass of 2 mmol ReCl_3_ (including 1 mmol YbCl_3_·6H_2_O and 1 mmol YCl_3_·6H_2_O), 14 mL of oleic acid and 30 mL of 1-octadecene were used as raw materials. Under the protection of high-purity argon, the temperature was raised to 160 °C and kept at this temperature for 60 min. After the reactant was cooled to room temperature, a methanol solution dissolved with 8 mmol NHF_4_ and 5 mmol NaOH was added dropwise. After stirring at room temperature for 30 min, the reactant was heated to 100 °C and incubated for a period of time so that the methanol solution in the reactant could be fully evaporated, then the reactant was heated to 300 °C and kept at this temperature for 60 min, after the reactant cooled to room temperature, it was washed with ethanol and cyclohexane several times and dried for use. The prepared upconversion nanoparticles were used as the core. During the encapsulation process, 0.5 mmol of ReCl_3_ (including YCl_3_·6H_2_O and HoCl_3_) was added to the prepared core dissolved in 3 mL of cyclohexane after the reaction was completed at 160 °C for 60 min. The mixture was stirred at room temperature for 20 min, then added to a methanol solution dissolved in 2 mmol NHF_4_ and 1.25 mmol NaOH. After stirring at room temperature for 30 min, the reactant was heated to 100 °C and kept for a period of time, so that the methanol solution in the reactant could be fully evaporated, and then the reactant was heated to 300 °C and kept at this temperature for 60 min. After the reactant was cooled to room temperature, it was washed several times with ethanol and cyclohexane, and dried for later use.

#### 2.2.2. Preparation of NaYF_4_:Yb,Tm UCNPs

The preparation of NaYF_4_:Yb,Tm was similar to the nuclear layer structure of NaYF_4_:Yb@NaYF_4_:Ho. In a total mass of 2 mmol ReCl_3_ (including 1.6 mmol YCl_3_·6H_2_O, 0.36 mmol YbCl_3_·6H_2_O and 0.04 mmol TmCl_3_·6H_2_O), 14 mL of oleic acid and 30 mL of 1-octadecenc were added. The 1-octadecene was used as the raw material, and the temperature was raised to 160 °C under the protection of high-purity argon and kept for 60 min. After the reactant was cooled to room temperature, a methanol solution dissolved with 8 mmol NHF_4_ and 5 mmol NaOH was added dropwise, and stirred at room temperature for 30 min, the reactant was heated to 100 °C and kept at this temperature for a period of time, so that the methanol solution in the reactant could be fully evaporated, then the reactant was heated to 300 °C and kept at this temperature for 60 min. After this, the reactant was cooled to room temperature, and washed several times with ethanol and cyclohexane before a secondary drying process was used to preserve it for later use. 

#### 2.2.3. Preparation of NaYF_4_:Yb,Ho UCNPs

The synthesis of NaYF_4_:Yb,Ho UCNPs is similar to the preparation process of NaYF_4_:Yb,Tm UCNPs, changing the molar ratio of Y:Yb:Ho = 80:18:2.

### 2.3. Surface Modification of Upconversion Nanomaterials

Surface modification of oil-soluble upconversion materials was accomplished using the classical Stober method. Using sonication, we dispersed 20 mg of oil-soluble UCNPs in 60 mL of isopropanol, add 2.5 mL of ammonia water and 20 mL of water to the flask, and kept the mixture at 35 °C under vigorous stirring. Then, 20 mL of isopropanol solution containing 50 μL of TEOS was added dropwise to the mixture and the reaction was maintained for another 3 h. Then, a solution containing 30 mL of isopropanol and 200 μL of APTES was added dropwise to the suspension for 1 h. After the reaction, the product was aged at room temperature for 2 h, the precipitate was separated by centrifugation, washed three times with deionized water and ethanol, and dried in an oven at 60 °C for 12 h to obtain amino-modified NaYF_4_:Yb@NaYF_4_:Ho and NaYF_4_:Yb,Tm UCNP.

### 2.4. Preparation of Capture Probes and Signal Probes

The signal probe was prepared using the classic glutaraldehyde cross-linking method. 10 mg of the upconversion material was dissolved in 5 mL of 10 mmol/L PBS (phosphate buffer saline) solution for ultrasonic dispersion for 30 min, and 1.25 mL of 25% glutaraldehyde solution and 100 mg of sodium borohydride were added at room temperature. After the reaction was completed, the precipitate was collected by centrifugation. The solid powder was washed three times with PBS, resuspended in 5 mL of 10 mmol/L PBS for ultrasonic dispersion, and a certain amount of antibody was added to be slowly shaken at room temperature for 6 h. The supernatant was collected by centrifugation at the same time, and the material was washed several times, mixed with 5 mL of 10 mmol/L PBS containing 3% BSA, and slowly shaken for 6 h at room temperature. After separation and washing, it was dispersed in 5 mL of PBS and stored at 4 °C.

Similarly, the capture probe was prepared by dissolving 10 mg Fe_3_O_4_ magnetic microspheres in 5 mL 10 mmol/L PBS solution. The operation method is similar to the preparation of the above signal probe. A certain amount of antigen was added and finally dispersed in 5 mL PBS buffer to obtain a capture probe with a concentration of 2 mg/mL.

### 2.5. Establishment of Fluorescence Immunoassay

In a typical experiment, 100 µL of IMI and CLO standards and 100 µL of capture probes were mixed in a 1.5 mL centrifuge tube, then a 200 µL of (each) signal probe was added to the mixture, and left to compete for 60 min. After the reaction, we collected the signal probe-capture probe complex through the external magnetic field, washed it with PBS three times, dispersed the precipitate in 500 µL PBS, and used the fluorescence spectrophotometer with an external 980 nm exciter to measure the fluorescence intensity values at 656 nm and 696 nm, respectively. After optimizing the pH value (5.0, 6.0, 7.4, 8.0, 9.0), Na^+^ concentration (10, 20, 30, 40, 50 mmol/L) and methanol concentration (0%, 2.5%, 5%, 10%) of PBS buffer solution in the competitive reaction system, the standard curve was established by changing the competitive reaction time. Finally, the optimal pH value, ion concentration, methanol content and competitive reaction time of the buffer solution were determined by F_max_/IC_50_ value. (F_max_ is the maximum value of fluorescence intensity, and IC_50_ is the corresponding standard concentration when the inhibition rate is 50%, i.e., sensitivity.)

### 2.6. Specificity

In order to investigate the specificity of the method, five other structural analogs to IMI and CLO were selected, i.e., acetamiprid, thiacloprid, nitenpyram, dinotefuran, and thiamethoxam. Acetamiprid, thiacloprid, nitenpyram, dinotefuran, thiamethoxam and IMI, CLO all belong to neonicotinoid pesticides and are structural analogs, and their application scope is also similar. Therefore, these pesticides were selected as specific research objects. Their standards were formulated at a concentration of 10 µg/L to cross-react with the capture probe signal probes. The fluorescence intensity values at emission wavelengths of 656 nm and 696 nm were recorded as I values. Only the fluorescence intensity values of the capture probe and the signal probe were recorded as I_0_, and ΔI = I_0_ − I was calculated to evaluate the specificity of the method.

### 2.7. Sample Handling

In order to further evaluate the detection accuracy and practical applicability of IMI and CLO by fluorescence immunoassay, an additive recovery experiment was carried out. The coefficient of variation (CV) was used as the basis for judging the precision of this method and each concentration was repeated 5 times. In this study, six fruit and vegetable samples were purchased from the market, including apples, oranges, peaches, cucumbers, tomatoes, and peppers. The extracted fruit and vegetable samples were pre-treated, 2 g of edible parts (accurate to 0.01 g) were weighed into a 50 mL centrifuge tube, 4 mL acetonitrile was added, and a high-speed tissue masher was used. Then, we homogenized an extract at 15,000 rpm for 1 min, added 1 g of sodium chloride, then homogenized an extract for 1 min, centrifuged at 3000 rpm for 5 min, took 2 mL of the supernatant into a 50 mL round-bottom flask, and rotated at 38 °C to dryness added 1 mL of 25% acetonitrile, sonicate until fully dissolved, and passed through a 0.22 µm filter.

### 2.8. Recovery Experiment

To perform the recovery experiment, we added 2, 20 and 200 ng/mL IMI and CLO standard solutions to the blank samples of pepper and cucumber, respectively, and added 5, 50 and 500 ng/mL IMI and CLO standard solutions to the blank samples of tomato, apple, orange and peach, respectively. After mixing, we treated them with the above sample treatment methods. Finally, we diluted them with PBS appropriately, and used this experimental method for detection.

### 2.9. HPLC Comparison Test

The chromatographic conditions of HPLC are as follows: chromatographic column: Bridge C18 (5 µm, 4.6 × 150 mm); mobile phase: methanol-water (32:68); flow rate 1.0 mL/min; column temperature: 30 °C; detection wavelength: 270 nm; injection volume: 20 µL.

The edible part of the fruit and vegetable samples was divided into two parts, and equal amounts of standard substances were added, respectively. One part was subjected to sample pretreatment as described in Section 2.6 and tested by this experimental method; the other part refers to GB/T 20769-2008 [27]. After the sample pretreatment, HPLC was used for detection, that is, after the sample was extracted with acetonitrile, salted out, centrifuged, and rotary evaporated, it was purified by a solid-phase extraction column and eluted with acetonitrile and toluene (3+1). After rotary evaporation, it was dried with nitrogen, and then acetonitrile decahydrate (3+2) solution was quickly added to mix, well-filtered through a 0.22 µm filter membrane, and then detected by HPLC.

### 2.10. Actual Sample Detection

The samples were selected from the local market (Tianjin, China), three each of peppers, cucumbers, tomatoes, oranges, peaches and apples, a total of 18 blank samples, numbered 1–18#, randomly added IMI and CLO standards to make blind samples, for the detection of immunoassay and HPLC in this experiment.

## 3. Results and Discussion

### 3.1. Characterization of the Prepared UCNPs

**Figure 1:**
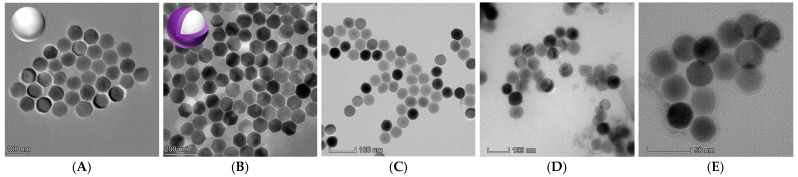
Transmission electron microscopy imaging of (**A**) oil-soluble core NaYF_4_:Yb UCNPs; (**B**) oil-soluble core-shell NaYF_4_:Yb@NaYF_4_:Ho UCNPs; (**C**) oil-soluble bare core NaYF_4_:Yb,Tm UCNPs; (**D**) water-soluble core-shell NaYF_4_:Yb@NaYF_4_:Ho UCNPs; (**E**) water-soluble naked core structure NaYF_4_:Yb,Tm UCNPs.

The morphologies of upconversion nanomaterials with oil-soluble and water-soluble NaYF_4_:Yb@NaYF_4_:Ho core-shell structure and NaYF_4_:Yb, Tm monolayer structure were characterized by transmission electron microscopy. The results are shown in the figure: TEM images show that the two synthesized oil-soluble upconversion materials are hexagonal phases with smooth surfaces, uniform size and good dispersion, among which the NaYF4:Yb@NaYF4:Ho (Figure 1B) core-shell structure particles. NaYF_4_:Yb UCNPs (Figure 1A) are the core of oil-soluble core-shell NaYF_4_:Yb@NaYF_4_:Ho UCNPs. The particle size of NaYF_4_:Yb UCNPs is about 75 nm and the crystal form is in a hexagonal phase, but partially spherical. A shell containing the rare earth element Ho was coated on the surface of NaYF_4_: Yb UCNPs to obtain NaYF_4_:Yb@NaYF_4_:Ho UCNPs. The particle size of NaYF_4_:Yb@NaYF_4_:Ho UCNPs is about 80nm, and the crystal form is in a standard β-hexagonal phase. It can be seen that this paper successfully coated a shell on the surface of NaYF_4_:Yb UCNPs by thermal decomposition method to form NaYF_4_:Yb@NaYF_4_:Ho UCNPs. The particle size of NaYF_4_:Yb,Tm (Figure 1C) monolayer structure is about 60–70 nm. The surface-modified upconversion materials (Figure 1D,E) are spherical particles with uniform particle size, and the surface is coated with a layer of silicon shell with a thickness of about 10 nm, which has good dispersibility in the water system. Oil-soluble nanoparticles were transformed into water-soluble nanoparticles by coating silicon shells on the surface of UCNPs, and their biocompatibility was improved by modifying amino groups on their surface.

**Figure 2:**
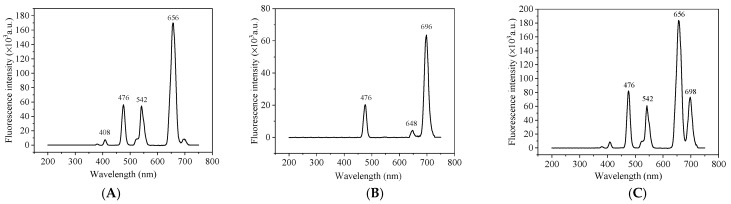
The fluorescence spectrum of (**A**) NaYF_4_:Yb@NaYF_4_:Ho; (**B**) NaYF_4_:Yb,Tm and (**C**) the mixture of NaYF_4_:Yb@NaYF_4_:Ho and NaYF_4_:Yb,Tm UNCPs under 980 nm excitation.

Under an 980 nm excitation light, NaYF_4_:Yb@NaYF_4_:Ho core-shell UCNPs produced four emission peaks at 408, 476, 542 and 656 nm, respectively, and the largest characteristic emission peak was at 656 nm (Figure 2A). NaYF_4_:Yb,Tm upconversion nanomaterials with bare core structure produced three emission peaks at 476, 648 and 696 nm, respectively, and 696 nm was the largest characteristic emission peak (Figure 2B). After mixing the above two materials, the emission peaks at 656 nm and 696 nm are independent and do not overlap, which can realize the simultaneous detection of two targets (Figure 2C). Through horizontal comparison, it is not difficult to find that the fluorescence intensity of core-shell structure is an order of magnitude higher than that of single-layer structure upconversion materials. This shows that core-shell upconversion materials can effectively reduce the quenching effect of surface defects such as hanging bonds and polymer vibrational groups on the luminescence of nanomaterials. It can effectively improve the luminous efficiency of the material, to further enhance the fluorescence. In this paper, Yb/Ho system was studied, and the proportion of doped rare earth elements was optimized in the synthesis of core-shell UCNPs. We adjusted the emission wavelength of NaYF_4_:Yb@NaYF_4_:Ho and enhanced its emission light after 600 nm, and widenened the application of this material in the fields of biological imaging, detection and multi-color upconversion.

**Figure 3:**
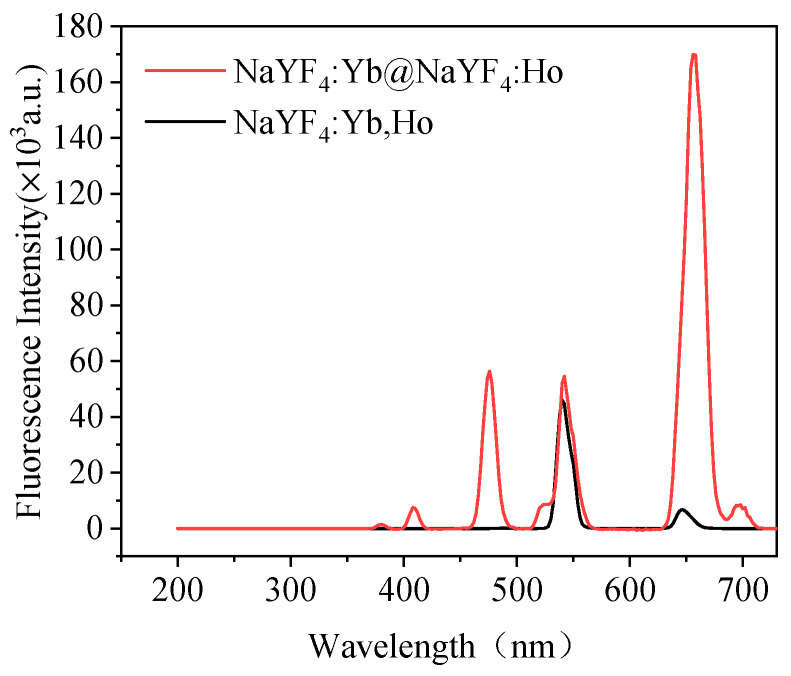
The fluorescence spectrum of NaYF_4_:Yb@NaYF_4_:Ho UCNPs and NaYF_4_:Yb,Ho UCNPs.

Under 980 nm excitation, the core-shell structure UCNPs NaYF_4_:Yb@NaYF_4_:Ho and monolayer UCNPs NaYF_4_:Yb, Ho have two peaks at 540 nm (green light) and 656 nm (red light). The fluorescence intensity ratios of core-shell structure and monolayer structure UCNPs at 540 nm:656 nm are about 1:3 and 13:1. Therefore, NaYF_4_:Yb@NaYF_4_:Ho core-shell UCNPs mainly emit red light, while NaYF_4_:Yb,Ho single-layer structure UCNPs mainly emit green light (Figure 3). Therefore, it can be shown that accurate upconversion luminescence color regulation can be achieved by rationally distributing the spatial position and relative concentration of activator ions. Separating Ho and Yb in NaYF_4_:Yb, Ho nanoparticles and Yb:Ho = 50:1 can suppress the luminescence at 540 nm and enhance the luminescence at 656 nm, making the Yb, Ho co-doped NaYF_4_ change from green to red.

**Figure 4:**
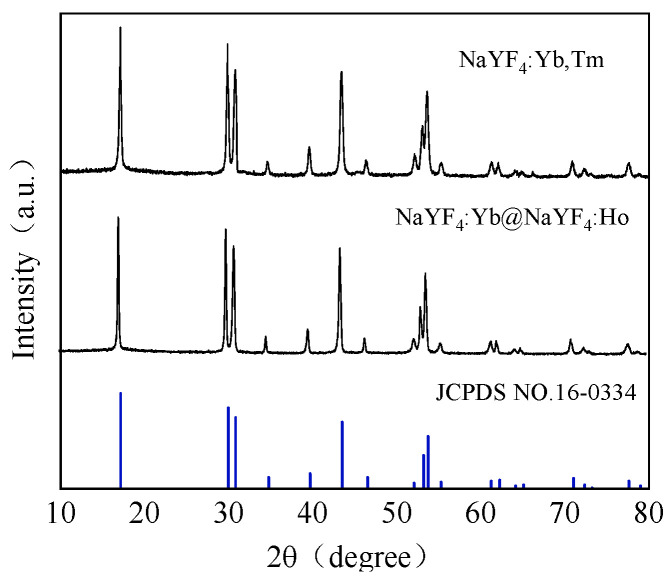
X-ray diffraction patterns of NaYF_4_:Yb@NaYF_4_:Ho UCNPs and NaYF_4_:Yb,Ho UCNPs.

The crystal structure and phase purity were determined by powder X-ray diffraction. The NaYF_4_:Yb@NaYF_4_:Ho and NaYF_4_:Yb,Tm upconversion nanomaterials were identified by X-ray diffraction as shown in Figure 4. The positions of all diffraction peaks and the relative intensities match well with the diffraction patterns of standard hexagonal β-phase UCNPs (JCPDS No. 16-0334). There are no diffraction peaks of other impurities indicating that the as-prepared NaYF_4_:Yb@NaYF_4_:Ho and NaYF_4_:Yb,Tm upconversion nanoparticles are well crystallized and exhibit good photoluminescence effectiveness.

**Figure 5:**
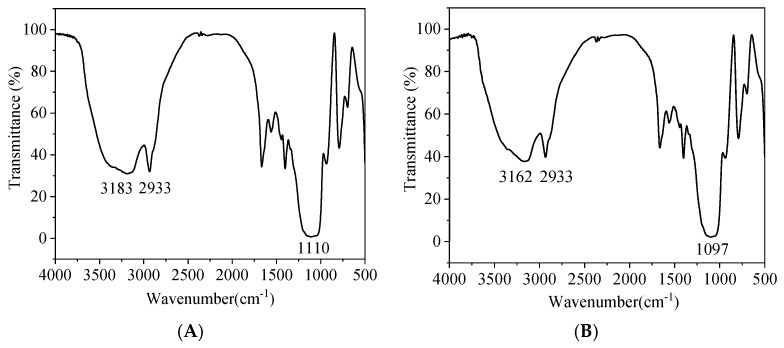
(**A**) Fourier transform infrared spectra of water-soluble NaYF_4_:Yb@NaYF_4_:Ho UCNPs; (**B**) and NaYF_4_:Yb,Tm UCNPs.

For the NaYF_4_:Yb@NaYF_4_:Ho UCNPs (Figure 5A), the symmetric stretching vibration of Si-O appeared in the region of about 1100 cm^−1^, which indicated that the surface of UCNP is coated with a silicon shell. The stretching and bending vibrations of amino groups appear at 3183 cm^−1^. The peak at 2933 cm^−1^ belongs to the asymmetric and symmetric stretching vibrations of the methylene group. Similar results were also observed in the infrared spectra of NaYF_4_:Yb,Tm (Figure 5B). For the NaYF_4_:Yb,Tm UCNPs, the symmetric stretching vibration of Si-O appeared in the region of 1097 cm^−1^, which shows that the material has been coated with a silicon shell. The stretching and bending vibration of the amino group appears at 3162 cm^−1^, indicating that the surface of the material has been modified with the amino group. Fourier transform infrared spectroscopy results show that both materials have been coated with silicon shells and modified with amino groups. The modified UCNPs have good water solubility and are conducive to binding with antibodies in immunoassay. The modified UCNPs have good dispersibility in the water system, no significant change in morphology, uniform particle size, and a layer of silicon shell with a thickness of about 5 nm is wrapped on the surface.

### 3.2. Optimize Probe Preparation Conditions

**Figure 6:**
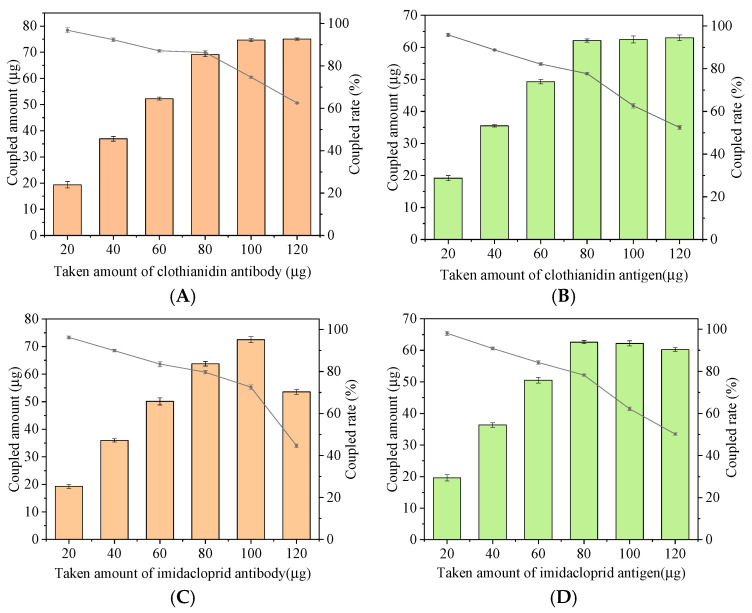
Optimizing the loading of Ab and Ag on signal (pink) and capture (green) probes. Optimizing the loading of (**A**) CLO antibody; (**B**) CLO antigen; (**C**) IMI antibody; (**D**) IMI antigen.

The preparation conditions of signal probes and capture probes directly affect the sensitivity and accuracy of fluorescent immunoassays. The BCA protein concentration assay kit was used to detect the coupling amount of antibody and coated antigen on the surface of UCNPs and MNPs. As shown in Figure 6A, when 5 mL of 2 mg/mL modified water-soluble UCNPs were immobilized, when the amount of antibody added was less than 80 µg, the coupling amount increased with the increase of the amount of antibody added, and the coupling rate decreased with the increase of the added amount. When the addition amount is greater than 80 µg, the coupling amount remains unchanged, and the coupling rate shows a significant downward trend, indicating that the amount of antibody coupled on the surface of the material has reached the saturation state, and excessive addition will cause waste. Therefore, 80 µg of CLO antibody was added to prepare a signal probe, and the coupling rate was 86.4%. At the same time, 5 mL of 2 mg/mL activated MNPs were fixed, and different amounts of the coating were added. According to Figure 6B, 80 μg of CLO antigen was finally added to prepare a capture probe, and the coupling rate was 77.7%. Similarly, the optimal dosage of IMI antibody (Figure 6C) was determined to be 100 µg, and the coupling rate was 72.5%; the optimum dosage of IMI antigen (Figure 6D) was 80 µg, and the coupling rate was 78.2%.

**Figure 7:**
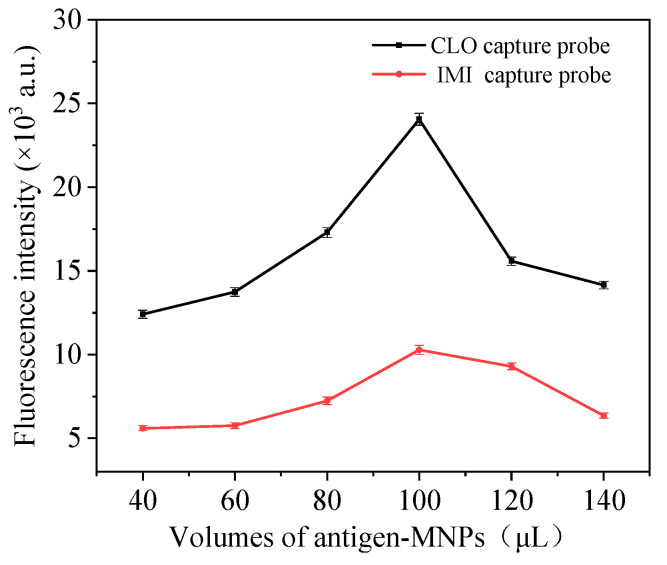
Optimization of the amount of capture probe added.

By changing the ratio of the signal probe and capture probe, the addition amount of the capture probe is optimized to obtain the best binding addition amount of antigen and antibody. In this experiment, the addition amount of both signal probes is 200 µL. The volumes of the two capture probes range from 40 to 140 µL. After the reaction, the fluorescence value is measured by a fluorescence spectrophotometer. According to the changing trend of fluorescence value (Figure 7), the fluorescence intensity of the reaction system increases with the increase of the addition of IMI and CLO capture probes. When the addition of both capture probes is 100 µL, the fluorescence intensity reaches the maximum. Increasing the amount of capture probe will lead to the darker color of the reaction system, cause background interference and reduce the fluorescence intensity. Therefore, the best addition amount of IMI and CLO capture probe is 100 µL.

### 3.3. Optimization of Method Conditions

The IC_50_ of this experimental method is the sensitivity. In order to achieve the best sensitivity, the parameters of the competition system were optimized: (1) pH value of buffer solution (2) Na^+^ concentration of buffer solution (3) methanol content. Taking IC_50_ and F_max_/IC_50_ as the evaluation criteria, the minimum value of IC_50_ and the maximum value of F_max_/IC_50_ were taken as the optimal conditions.

**Figure 8:**
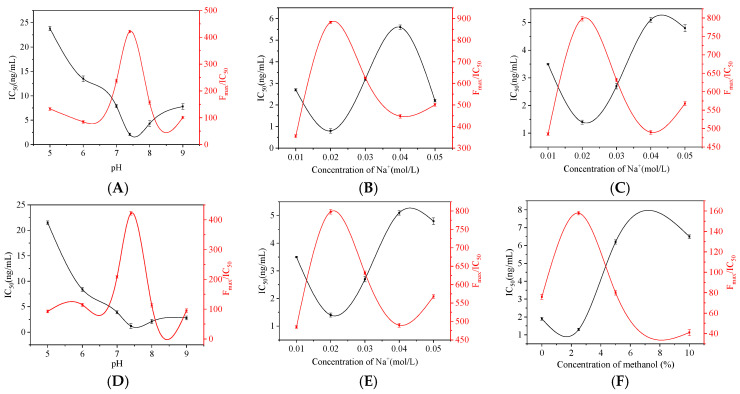
Optimization of pH, Na+ concentration, methanol content of (**A**–**C**) IMI and (**D**–**F**) CLO.

Under the condition of immune reaction, the sensitivity of immune reaction will be affected by over acid or over alkali environment. Adjust the pH value in the system to 5.0, 6.0, 7.4, 8.0 and 9.0. As shown in Figure 8A,D, IC_50_ first decreased and then increased with the increase of pH value. When the pH value was 7.4, the IC_50_ value was the lowest, while F_max_/IC_50_ reached the highest value. Therefore, pH 7.4 was selected as the optimal reaction system. The ion concentration in the buffer, i.e., Na^+^ concentration, will also interfere with the results. Set the Na^+^ concentration to five concentrations: 0.01, 0.02, 0.03, 0.04 and 0.05 mol/L. With the increase of Na^+^ concentration in the buffer (Figure 8B,E), the IC_50_ of IMI and CLO showed a trend from decreasing to increasing and then decreasing. When the Na^+^ concentration was 0.02 mol/L, IC_50_ reached the lowest, while F_max_/IC_50_ was the highest, and the Na^+^ concentration of the final selection buffer was 0.02 mol/L. In order to improve the solubility of the target in the aqueous phase, a certain amount of methanol was added to the system. Set the methanol content to 0%, 2.5%, 5%, 10%. The results are shown in Figure 8C, F, where IC_50_ increased significantly with the increase of methanol content, and F_max_/IC_50_ showed a downward trend. When the methanol content was 2.5%, the IC_50_ value reached the lowest value, while the F_max_/IC_50_ value reached the highest value. The methanol content of the final selection buffer was 2.5%.

### 3.4. Determination of the Detection Limit of the Method

**Figure 9:**
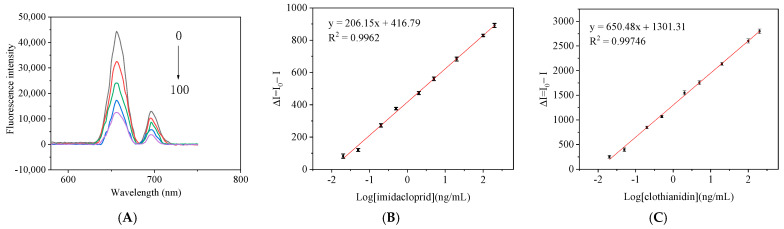
(**A**) Upconversion fluorescence spectra of UCNPs as a function of IMI or CLO concentrations; linear relationship between upconverted fluorescence intensity with (**B**) IMI and (**C**) CLO concentration.

In this study, the characteristic absorption peak at 656 nm of NaYF_4_:Yb@NaYF_4_:Ho UCNPs was used as the detection wavelength of CLO, and the characteristic absorption peak at 696 nm of NaYF_4_:Yb,Tm UCNPs was used as the detection wavelength of IMI. An upconversion magnetic separation fluorescence immunoassay method for the simultaneous detection of IMI and CLO was established. Under the best competitive reaction conditions, the standard solutions of different concentrations of IMI and CLO were mixed with a 100 μL capture probe in a 1.5 mL centrifuge tube. Then, 200 μL signal probes were added and reacted at room temperature for 1 h. After the reaction, it was separated by the external magnetic field and washed with PBS 3 times. Under the optimal competitive reaction conditions, the change in fluorescence intensity ΔI (ΔI = I_0_ − I; I_0_ and I represent the fluorescence intensity in the absence and presence of analytes, respectively) and the logarithm of the concentrations of IMI and CLO (Figure 9A). The limit of detection (LOD) and sensitivity (IC_50_) of IMI was 0.032 ng/mL and 4.7 ng/mL, respectively, and the linear range (IC_10_–IC_90_) was 0.032–285.75 ng/mL (Figure 9B). The LOD and the IC_50_ of CLO were 0.028 ng/mL and 2.1 ng/mL, and the linear range was 0.028–200 ng/mL (Figure 9C). Tao Zhexuan [28] established a multicolor upconversion immunoassay method for the detection of IMI and thiacloprid by synthesizing NaYF_4_:Yb,Er UCNPs and NaYF_4_:Yb,Tm UCNPs. The LOD of IMI was 0.32 ng/mL and IC_50_ was 5.8 ng/mL; LOD of thiacloprid was 0.61 ng/mL and IC_50_ was 6.45 ng/mL. Compared with Tao’s [28] experimental method, he synthesized NaYF_4_:Yb,Er UCNPs and NaYF_4_:Yb,Tm UCNPs by hydrothermal method, this experiment synthesized NaYF_4_:Yb,Tm UCNPs and NaYF_4_:Yb@NaYF_4_:Ho UCNPs have higher fluorescence intensity by thermal decomposition method. Applying these two UCNPs to the upconversion immunoassay method for the simultaneous detection of IMI and CLO has the advantages of lower detection limit and higher sensitivity, which can provide technical support for the detection of neonicotinoids pesticides.

### 3.5. Specificity

**Figure 10:**
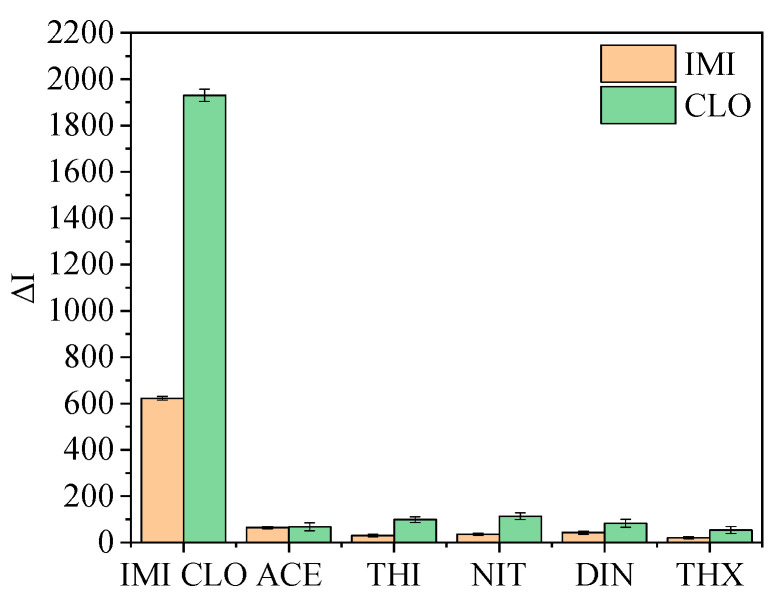
Method specificity evaluation (The concentrations of all standards are 10 µg/L).

In order to evaluate the specificity of the fluorescence immunoassay method, 10 µg/L IMI, CLO and their structural analogs were added to the reaction system for detection, and the corresponding fluorescence intensity difference was calculated according to the blank fluorescence intensity (Figure 10). When IMI and CLO were added, the fluorescence changed significantly, indicating that the UCNPs were bound with the antibody specifically binding to IMI and CLO. When the ACE, THI, NIT, DIN, THX and other neonicotinoids pesticide structural analogs were added, it only resulted in some nonspecific adsorption. There was almost no specific reaction with the antibody on the UCNPs. Therefore, the results show that this method has high specificity.

### 3.6. Elimination of Matrix Effects

**Figure 11:**
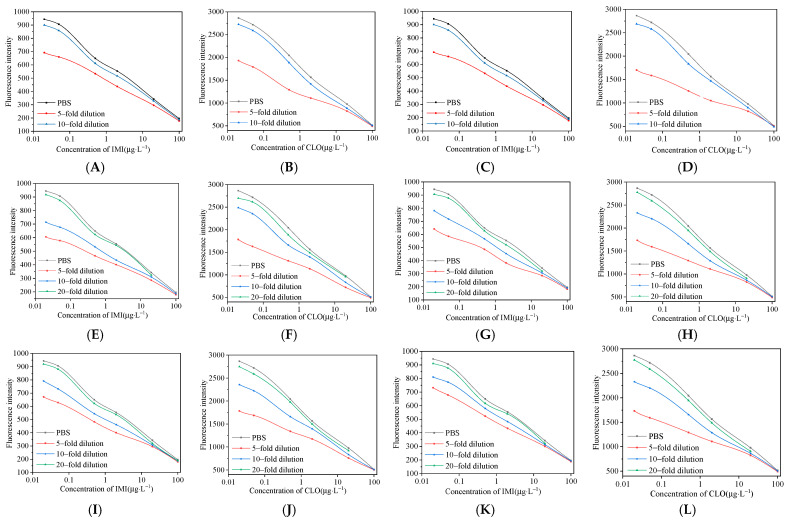
Evaluation of matrix effect in fruit and vegetable acetonitrile extracts; (**A**,**B**) cucumber; (**C**,**D**) pepper; (**E**,**F**) apple; (**G**,**H**) orange; (**I**,**J**) peach; (**K**,**L**) tomato.

The vegetable and fruit samples used in this experiment will cause certain interference to the detection results due to the influence of sugars, pigments and other matrices. After extracting the six vegetable and fruit samples of apple, orange, peach, cucumber, tomato and pepper with acetonitrile, the method of buffer dilution was used to eliminate the influence of the matrix. It can be seen from Figure 11 that the curves for cucumber and pepper diluted with PBS 10 times and apple, orange, peach and tomato diluted with PBS 20 times basically coincide with the standard curve, indicating that this method can effectively eliminate matrix interference.

### 3.7. Spiked Recovery Experimental Results

IMI and CLO standards with low, medium and high concentrations were added to apple, orange, peach, cucumber, tomato, and pepper, respectively, and five parallel groups were set for the determination of each concentration of each sample. As shown in Table 1, the recoveries of IMI and CLO were 83.33–115.02% and 84.49–106.64%, respectively, and the coefficient of variation was less than 10% for both. In apple, orange, peach, cucumber, tomato and pepper samples, the detection limit of this method was 5 ng/mL, 2 ng/mL, 5 ng/mL, 5 ng/mL, 5 ng/mL, and 5 ng/mL, respectively, which were much lower than those in China Maximum Residue Limit Standards.

### 3.8. Correlation of Immunoassays with HPLC

**Figure 12:**
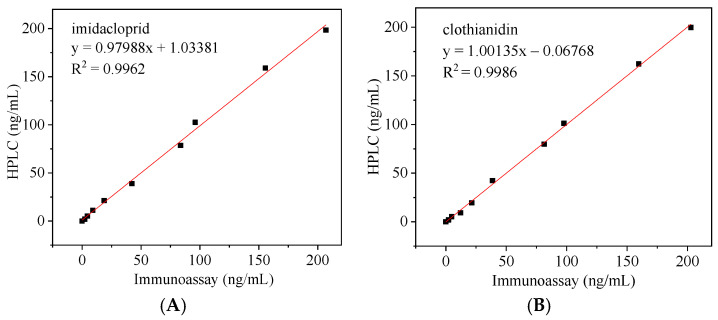
Correlation between HPLC and the method of this study for the detection of (**A**) IMI and (**B**) CLO.

The concentrations of IMI and CLO in cucumber were determined using this experimental immunoassay and HPLC. As shown in Figure 12, the correlation equations for IMI and CLO are y = 0.97988x + 1.03381 (R^2^ = 0.9962) and y = 1.00135x − 0.06768 (R^2^ = 0.9986), respectively. The results show a good correlation between the two methods, and the immunoassay in this experiment is accurate and reliable and can be used to detect IMI and CLO in real samples. The retention times of IMI and CLO were 11.5 and 18 min, respectively.

### 3.9. Test Results of Actual Samples

A total of 18 blank samples of 3 peppers, cucumbers, tomatoes, oranges, peaches and apples, were numbered 1–18#. They were randomly added with IMI and CLO standards to make blind samples for immunoassay and HPLC methods. The results are shown in Table 2: Six IMI positive samples were finally detected, including one pepper, one cucumber, one tomato, one orange, one peach and one apple. There were 6 CLO positive samples, including 2 cucumbers, 1 orange, 1 peach and 2 apples. In order to further evaluate the detection accuracy and practical applicability of the fluorescent immunoassay method in this experiment, the detection of IMI and CLO in actual samples was carried out. The results show that in Table 2, the detection results of the established fluorescence immunoassay method are in good agreement with the HPLC detection results. This result can be used for the determination of actual samples.

## 4. Conclusions

In this paper, a novel core-shell structure material is applied to the analysis of immune detection for the first time. Based on the different emission spectra of Yb/Ho and Yb/Tm doped NaYF_4_ UCNPs excited by a 980 nm laser, a dual-signal probe is prepared. The magnetic nanoparticle trapping probe can realize the principle of rapid separation of immune complexes from the test system. An upconversion-magnetic separation fluorescence immunoassay was successfully developed for simultaneous detection of IMI and CLO. This method has the advantage of causing a rapid, simple, sensitive, innovative transformation of the core-shell nanoparticles coupling antibody, compared to a traditional transformation of materials with higher luminous efficiency and gain better detection results in fluorescence immunoassay detection, proving that the material in the field of food safety testing particularly multicolor code has very strong potential applications. These results provide a new idea for the application of upconversion materials in immunofluorescence in the future.

## Figures and Tables

**Table 1:** Average recoveries of IMI and CLO spiked standards.

Sample	Scalar (μg/kg)	IMI			CLO		
		Mean ± SD (μg/kg)	Recovery Rate (%)	Coefficient of Variation (%)	Mean ± SD (μg/kg)	Recovery Rate (%)	Coefficient of Variation (%)
pepper	2	1.71 ± 0.15	85.34	8.6	1.99 ± 0.18	99.28	9.0
	20	20.72 ± 1.43	103.58	6.9	21.33 ± 1.30	106.64	6.1
	200	182.50 ± 7.85	91.25	4.3	168.98 ± 6.08	84.49	3.6
cucumber	2	1.90 ± 0.17	95.10	9.2	1.93 ± 0.17	96.27	8.9
	20	17.98 ± 0.91	89.89	5.1	20.55 ± 0.97	102.75	4.7
	200	180.47 ± 3.43	90.24	1.9	175.94 ± 4.57	87.97	2.6
tomato	5	4.29 ± 0.35	85.74	8.1	5.05 ± 0.35	100.93	6.9
	50	47.11 ± 2.12	94.23	4.5	43.62 ± 1.57	87.24	3.6
	500	480.43 ± 11.53	96.09	2.4	444.02 ± 7.99	88.80	1.8
orange	5	4.17 ± 0.30	83.33	7.2	4.65 ± 0.35	92.94	7.5
	50	50.15 ± 2.81	100.31	5.6	50.38 ± 1.66	100.76	3.3
	500	425.36 ± 13.19	85.07	3.1	476.60 ± 7.63	95.32	1.6
peach	5	4.31 ± 0.29	86.12	6.8	4.37 ± 0.27	87.48	6.2
	50	47.96 ± 1.97	95.92	4.1	49.18 ± 1.67	98.36	3.4
	500	570.60 ± 15.41	114.12	2.7	489.07 ± 9.78	97.81	2.0
apple	5	4.40 ± 0.33	87.97	7.5	4.37 ± 0.34	87.36	7.7
	50	55.96 ± 2.80	111.91	5.0	49.69 ± 1.89	99.38	3.8
	500	575.08 ± 19.55	115.02	3.4	522.91 ± 6.27	104.58	1.2

**Table 2:** Detection of IMI and CLO in actual samples.

Sample Serial Number	This Experimental Method		HPLC	
	IMI (ng/mL)	CLO (ng/mL)	IMI (ng/mL)	CLO (ng/mL)
1#pepper	-	-	-	-
2#pepper	-	-	-	-
3#pepper	22.7	-	23.2	-
4#cucumber	-	82.4	-	85.2
5#cucumber	-	-	-	-
6#cucumber	61.8	103.4	63.7	101.1
7#tomato	92.1	-	94.8	-
8#tomato	-	-	-	-
9#tomato	-	-	-	-
10#orange	-	19.2	-	20.3
11#orange	136.4	-	132.8	-
12#orange	-	-	-	-
13#peach	31.6	54.7	30.2	55.8
14#peach	-	-	-	-
15#peach	-	-	-	-
16#apple	-	36.7	-	38.1
17#apple	48.7	-	47.9	-
18#apple	-	112.3	-	114.1

-: means not detected.

## Data Availability

Data is contained within the article.
